# Impact of EBUS-TBNA in addition to [^18^F]FDG-PET/CT imaging on target volume definition for radiochemotherapy in stage III NSCLC

**DOI:** 10.1007/s00259-021-05204-7

**Published:** 2021-02-05

**Authors:** Maja Guberina, Kaid Darwiche, Hubertus Hautzel, Till Ploenes, Christoph Pöttgen, Nika Guberina, Ken Herrmann, Lale Umutlu, Axel Wetter, Dirk Theegarten, Clemens Aigner, Wilfried Ernst Erich Eberhardt, Martin Schuler, Rüdiger Karpf-Wissel, Martin Stuschke

**Affiliations:** 1 Department of Radiotherapy, University Hospital Essen, West German Cancer Center, University Duisburg-Essen, Essen, Germany; 2 Department of Pulmonary Medicine, Section of Interventional Pneumology, University Medicine Essen – Ruhrlandklinik, West German Cancer Center, University Duisburg-Essen, Essen, Germany; 3 Department of Nuclear Medicine, University Hospital Essen, West German Cancer Center, University Duisburg-Essen, Essen, Germany; 4 German Cancer Consortium (DKTK), Partner Site University Hospital Essen, Essen, Germany; 5 Department of Thoracic Surgery and Thoracic Endoscopy, University Medicine Essen – Ruhrlandklinik, West German Cancer Center, University Hospital Essen, University Duisburg-Essen, Essen, Germany; 6 Institute of Diagnostic, Interventional Radiology and Neuroradiology, University Hospital Essen, University Duisburg-Essen, Essen, Germany; 7 Institute of Pathology, University Hospital Essen, West German Cancer Center, University Duisburg-Essen, Essen, Germany; 8 Department of Medical Oncology, University Hospital Essen, West German Cancer Center, University Duisburg-Essen, Essen, Germany; 9 Division of Thoracic Oncology, University Medicine Essen – Ruhrlandklinik, West German Cancer Center, University Duisburg-Essen, Essen, Germany

**Keywords:** Non-small cell lung cancer (NSCLC), Stage III, [^18^F]FDG-PET/CT, EBUS-TBNA, Radiotherapy, Target volume definition, Radiation

## Abstract

**Purpose/introduction:**

[^18^F]FDG-PET/CT is the standard imaging-technique for radiation treatment (RT) planning in locally advanced non-small cell lung cancer (NSCLC). The purpose of this study was to examine the additional value of endobronchial-ultrasound transbronchial needle aspiration (EBUS-TBNA) to standard PET/CT for mediastinal lymph-node (LN) staging and its impact on clinical target volume (CTV).

**Materials and methods:**

All consecutive patients with primary stage III NSCLC who underwent [^18^F]FDG-PET/CT and EBUS-TBNA prior to RT were analyzed from 12/2011 to 06/2018. LN-stations were assessed by an expert-radiologist and a nuclear medicine-physician. CTV was evaluated by two independent radiation oncologists. LNs were grouped with increasing distance along the lymphatic chains from primary tumor into echelon-1 (ipsilateral hilum), echelon-2 (LN-station 7 and ipsilateral 4), and echelon-3 (remaining mediastinum and contralateral hilum).

**Results:**

A total of 675 LN-stations of which 291 were positive for tumor-cells, were sampled by EBUS-TBNA in 180 patients. The rate of EBUS-positive LNs was 43% among all sampled LNs. EBUS-positivity in EBUS-probed LNs decreased from 85.8% in echelon-1 LNs to 42.4%/ 9.6% in echelon-2/ -3 LNs, respectively (*p* < 0.0001, Fisher’s exact test). The false discovery rate of PET in comparison with EBUS results rose from 5.3% in echelon-1 to 32.9%/ 69.1% in echelon-2/ -3 LNs, respectively (*p* < 0.0001, Fisher’s exact test). Sensitivity and specificity of FDG-PET/CT ranged from 85 to 99% and 67 to 80% for the different echelons. In 22.2% patients, EBUS-TBNA finding triggered changes of the treated CTV, compared with contouring algorithms based on FDG-avidity as the sole criterion for inclusion. CTV was enlarged in 6.7% patients due to EBUS-positivity in PET-negative LN-station and reduced in 15.5% by exclusion of an EBUS-negative but PET-positive LN-station.

**Conclusion:**

The false discovery rate of [^18^F]FDG-PET/CT increased markedly with distance from the primary tumor. Inclusion of systematic mediastinal LN mapping by EBUS-TBNA in addition to PET/CT has the potential to increase accuracy of target volume definition, particularly in echelon-3 LNs. EBUS-TBNA is recommended as integral part of staging for radiochemotherapy in stage III NSCLC.

**Supplementary Information:**

The online version contains supplementary material available at 10.1007/s00259-021-05204-7.

## Introduction

Consensus on radiotherapy planning recommends the inclusion of [^18^F]FDG-PET-positive lymph nodes in the clinical target volume (CTV) for irradiation of stage III non-small cell lung cancer (NSCLC) as a main criterion [1, 2]. Major prospective trials, such as RTOG 0617 and PET-Plan trial, established PET-based target volumes as a de facto standard for definitive radiochemotherapy planning [3, 4]. The PET-Plan trial demonstrated equivalence in terms of locoregional tumor control between an [^18^F]FDG-PET-based planning arm, using the PET-positivity as the single dominant criterion for delineation of the radiation target, compared with a “conventional target” arm. The conventional arm incorporated treatment fields based on PET as well as on CT information, including locoregional elective lymph node irradiation. In both arms histopathologically, positive lymph node (LN) stations were included in the CTV. Nevertheless, a not to be underestimated hazard ratio for overall survival in this trial was in favor of the conventional treatment arm in an intent-to-treat analysis (HR = 1.24 (95% CI: 0.86–1.90)).

Five-year survival rates approach 32–44% with definitive radiochemotherapy as well as with neoadjuvant radiochemotherapy and surgery for stage III NSCLC [3, 5]. The vast majority of patients received an [^18^F]FDG-PET/CT in the latter trials for staging [6, 7].

However, [^18^F]FDG-PET/CT alone is not an optimal diagnostic procedure to differentiate between malignant N0-N1 status and mediastinal N2-N3 LN-involvement. In their Cochrane review, Schmidt-Hansen et al. estimated the sensitivity and specificity of [^18^F]FDG-PET to be 81.3 and 79.4%, respectively, using a maximum standardized uptake value (SUV_max_) greater than 2.5 in a lesion as a PET-positivity criterion [8].

Interdisciplinary guidelines advocate invasive mediastinal staging using EBUS-TBNA or mediastinoscopy for patients with suspected or proven NSCLC without distant metastases and with PET-positive mediastinal lymph nodes. The primary aim here is to avoid exclusion of patients from upfront surgery due to false-positive PET/CT results [9–11]. Usually, EBUS-TBNA as the least invasive method is performed as the initial diagnostic procedure. In the case of negative EBUS-TBNA subsequent surgical staging by mediastinoscopy or video-assisted mediastinal lymph node sampling is performed to confirm the negative result. For patients receiving definitive radiochemotherapy, false-positive PET-findings might increase the target volume. A histopathological verification of PET findings might enable an accurate target volume definition prior treatment avoiding unnecessary normal tissue exposure.

The aim of the present study was to analyze the prevalence and false discovery rate of malignant LNs by [^18^F]FDG-PET compared with EBUS-TBNA in stage III NSCLC patients, receiving neoadjuvant or definitive radiochemotherapy, depending on the distance of the lymph node station from the primary tumor.

In addition, we also aimed to quantify the impact of EBUS-TBNA on the clinically applied target volume compared with delineation based solely on PET/CT by analyzing the frequency of omission of PET-positive but EBUS-negative LN-stations from the CTV and inclusion of PET-negative but EBUS-positive LN-stations in the CTV. The change of the irradiation target volume was examined depending on the location of the lymph node stations in the mediastinum.

## Materials and methods

All consecutive stage III (AJCC/UICC/TNM 8^th^ edition) NSCLC patients presented in the Department of Radiotherapy at the West German Lung Cancer Center from December 2011 to June 2018 were analyzed in the retrospective study design. All study patients started neoadjuvant or definitive radiochemotherapy at this department with curative intent. Eligible patients were older than 18 years and had to have a histologically proven NSCLC. Compulsory inclusion criteria were EBUS-TBNA and [^18^F]FDG-PET/CT imaging for primary staging done prior to treatment.

Furthermore, histopathology other than NSCLC and patients with another simultaneous malignant tumor entity or with recurrent lung cancer along with prior treatment were excluded.

All patients provided informed consent to radiochemotherapy. This retrospective study was approved by the local ethics committee (Ethics Committee of the Medical Faculty of the University Duisburg-Essen, 19-9056-BO). Contrast-enhanced [^18^F]FDG-PET/CT imaging was performed on the PET/CT Biograph-mCT-scanner (Siemens Healthineers, Germany) approximately 60 minutes after intravenous injection of 250–400 MBq [^18^F]FDG-ligand complex. PET/CT images were analyzed by at least one expert radiologist and one nuclear medicine physician followed by a consensus reading in the case of discrepancy and finally graded on a 4-point Likert scale (1–4). Grades 3 and 4 (inflammation or malignancy, malignancy) were counted as positive [12].

EBUS-TBNA was performed in a systematic manner via a rigid bronchoscope under general anesthesia, assessing all LN-stations and sampling all recognizable LNs larger than 5 mm: LN-stations 10-12 L, 10-12R, 7, 4 L, 4R, 2 L, and 2R.

After the last assignment, follow-up visits were scheduled every 3 months for the first 2 years and every 6 months from the third to the fifth year. In the case of unclear or prominent findings, the time interval was shortened and the next follow-up was conducted within 6 weeks. Physical examination was mandatory and computed tomography recommended at each visit.

The CTV was delineated on an exhale phase of a 4D-CT, a prospectively gated exhale CT, or on a voluntary breath-hold CT-scan depending on whether free breathing, free breathing with gating, or voluntary breath-hold techniques for motion mitigation were used. The target volume definition was performed according to the outlines of the RTOG 0617 protocol [3]. LN-stations were defined on CT according to the IASLC lung cancer map [13] and the Japanese Society for Radiation Oncology atlas [14].

In addition, LN-stations along the lymphatic drainage chains [15] were grouped into echelon-1, -2, and -3 from the ipsilateral hilum as the first echelon (echelon-1) over the ipsilateral central mediastinum, i.e., LN-stations 7 and ipsilateral LN-station 4 as the second echelon (echelon-2), to the upper ipsilateral mediastinum at LN-station 2 or the contralateral mediastinal LN-stations 4 and 2 and including the contralateral hilum as the third echelon (echelon-3).

LN-stations 5 and 6 were not included in this analysis, as they were not routinely accessible to EBUS-TBNA and in order to avoid false-positive findings based on this fact for PET in comparison to EBUS. In case of negative EBUS-TBNA where the suspicion of malignancy remains high, surgical staging was performed.

All patients were treated by dose delivery methods according to the state-of-the-art techniques available for the period of consideration using 6 or 8 MV photon beams. The treatment technique till early 2014 was predominantly 3D conformal radiotherapy. From 2015 up to now, intensity-modulated radiation therapy (IMRT) and volumetric modulated arc therapy (VMAT) with daily online cone beam imaging control were administered.

Statistical analysis was conducted using SAS software version 9.4, SAS/STAT 15.1 (SAS, Institute, Cary, NC). The procedures Logistic and Freq were used.

For calculation of the false discovery rate (FDR), the following formulae will apply [16]:

1 $$ \mathrm{FDR}=\frac{\mathrm{number}\ \mathrm{of}\ \mathrm{false}\ \mathrm{positives}}{\mathrm{number}\ \mathrm{of}\ \mathrm{true}\ \mathrm{positives}+\mathrm{false}\ \mathrm{positives}} $$

2 $$ \mathrm{FDR}=1-\frac{\mathrm{sensitivity}\ast \mathrm{prevalence}}{\mathrm{sensitivity}\ast \mathrm{prevalence}+\left(1-\mathrm{specificity}\right)\ast \left(1-\mathrm{prevalence}\right)} $$

For calculation of the false negative rate (FNR), the following formula applies:

$$ \mathrm{FNR}=\frac{\mathrm{number}\ \mathrm{of}\ \mathrm{false}\ \mathrm{negatives}}{\mathrm{number}\ \mathrm{of}\ \mathrm{false}\ \mathrm{negatives}+\mathrm{true}\ \mathrm{positives}} $$

FDR, FNR, as well as sensitivity, specificity and prevalence were calculated for two-way frequency tables of categorical variables. All test characteristics calculated for PET-positivity were related to EBUS results as the reference standard. The true prevalence of disease in the lymph node echelon of interest or mediastinum can be estimated from the EBUS-positivity rate divided by EBUS specificity, given the specificity of an EBUS-TBNA probe is about 100% [17]. Sensitivity of EBUS-TBNA is assumed to be at minimum 80%. The FDR of PET in comparison to the true lymph node positivity (FDR_PET_True_) is smaller than the FDR compared with EBUS positivity (FDR_PET_EBUS_):

3 $$ {\mathrm{FDR}}_{\mathrm{PET}\_\mathrm{True}}=\frac{{\mathrm{sensitivity}}_{\mathrm{EBUS}}-1+{\mathrm{FDR}}_{\mathrm{PET}\_\mathrm{EBUS}}}{{\mathrm{sensitivity}}_{\mathrm{EBUS}}}, $$

provided that sensitivity of EBUS is independent of the PET result (Eq. 3). Then, the number of PET-positive and truly involved lymph nodes (N_PET+_True+_) will be the number of PET-positive and EBUS-positive lymph nodes (N_PET+_EBUS+_) divided by sensitivity of EBUS:

$$ {\mathrm{N}}_{\mathrm{PET}+\_\mathrm{True}+}=\frac{{\mathrm{N}}_{\mathrm{PET}+\_\mathrm{EBUS}+}}{{\mathrm{sensitivity}}_{\mathrm{EBUS}}} $$

Equation 3 is finally derived from the following two equations:

$$ {\displaystyle \begin{array}{c}{\mathrm{FDR}}_{\mathrm{PET}\_\mathrm{True}}=1-\frac{{\mathrm{N}}_{\mathrm{PET}+\_\mathrm{True}+}}{\mathrm{numberofallPETpositivelymphnodes}}\\ {}\\ {}{\mathrm{FDR}}_{\mathrm{PET}\_\mathrm{EBUS}}=1-\frac{{\mathrm{N}}_{\mathrm{PET}+\_\mathrm{EBUS}+}}{\mathrm{numberofallPETpositivelymphnodes}}\end{array}} $$

All *p* values are provided for two-sided hypotheses.

## Results

A total of 180 patients fulfilled the inclusion criteria of this study. Patients’ characteristics are shown in Table 1. On a per patient basis, 109 patients had a positive EBUS-TBNA probe in mediastinal lymph nodes (Table 1). Of the 143 patients with PET-positive mediastinum, 10 patients were positive by PET in LN-stations 5/6 alone not accessible by EBUS-TBNA and also were EBUS-negative in other mediastinal LN-stations. Omitting these 10 patients and LN-stations 5/6 from the analysis, the false discovery rate of PET to detect mediastinal involvement in comparison to EBUS was 22.7% on a per patient basis. The respective sensitivity and specificity of PET was 93.6% and 50.8% in the remaining 170 patients.

**Table 1:** Patient characteristics

Patient characteristics	Number of Patients
Histology	
Adeno -Ca	83
Squamous Cell Ca	80
Other	17
cT-category	
T1	18
T2	35
T3	53
T4	74
cN-category	
cN2/N3 - positive by EBUS and positive by PET	102
cN2/N3 - negative by EBUS and positive by PET	30
cN2/N3 - positive by EBUS and negative by PET	7
UICC-stage	
IIIA	74
IIIB	80
IIIC	26
RT-intent	
Definitive RT/CTx	114
Neoadjuvant RT/CTx	66
Laterality of the primary tumor	
left-sided	83
right-side	93
bilateral	4
Age	Median and Range
Median	62.9 years
Range	43.6–84.0 years

Note: All numbers represent patients’ counts, except in the rows with patients’ age. cN2/ N3: involvement of echelon-2 or -3 LNs other than LN-stations 5/6, accessible by EBUS and PET

Table 2 depicts the results of all diagnostic or therapeutic procedures in the EBUS-TBNA probed hilar and mediastinal LN-stations. In total, 675 LN-stations were sampled by EBUS-TBNA in 180 patients, whereas 291 of them were histopathologically or cytopathologically positive for tumor cells (EBUS-positive). All of these LN-stations were also assessed in the nuclear medicine expert report and 377 LN-stations were PET-positive, 334 and 43 graded as Likert 4 and Likert 3, respectively, on a four-point Likert scale. In addition, the location of lymph nodes with respect to the CTV, included or excluded, was evaluated for all LN-stations.

**Table 2:** Number of assessed lymph node stations per diagnostic or therapeutic procedure

Procedure	Number of assessed LN-stations	LN-stations with positive findings	LN-stations with negative findings
EBUS-TBNA	675	291	384
Reported PET-Result	675	377	298
Coverage of the LN-station by the clinical target volume (CTV)	675	416	259

Note: All numbers represent lymph node station (LN-station) counts.

Cross-tabulation of EBUS- and PET-positivity in the 675 EBUS-sampled LN-stations is shown in Table 3. The rates of EBUS- and PET-positivity were 43.1 and 55.9%, respectively, in this group of patients. In comparison to EBUS-TBNA as a reference test, nuclear medicine report had a rather high overall false discovery rate of 27.1% and a high sensitivity of 94.5% per LN-station.

**Table 3:** Cross tabulation of results from EBUS-TBNA, PET-reports, and target volume coverage analysis from patients with locally advanced NSCLC, stage IIIA-IIIC, treated with definitive or neoadjuvant radiochemotherapy. *N* = 675 LN-stations in 180 patients

	Nuclear medicine report positive	Nuclear medicine report negative	Irradiated LN-stations	Not-irradiated LN-stations
EBUS-TBNA positive	275	16	289	2
EBUS-TBNA negative	102	282	127	257
Irradiated LN-station	338	78		
Not-irradiated LN-station	39	220		
Nuclear medicine report positive and EBUS-negative LN-stations			64	38
Nuclear medicine report negative and EBUS-positive LN-stations			15	1
Nuclear medicine report negative and EBUS-negative LN-stations			63	219

Note: All numbers represent lymph node station (LN-station) counts.

Table 4 shows the cross-tabulation of the LN-stations in echelons 1–3 with respect to PET-positivity/negativity as well as EBUS- positivity/negativity. The rate of EBUS-positivity was 85.8% per EBUS-probed LNs in echelon-1 LNs and decreased significantly to 42.4 and 9.6% in EBUS-probed echelon-2 and -3 LNs, respectively (*p* < 0.0001, Fisher’s exact test for comparison of all three echelons as well as echelon-1 with echelon-2 or echelon-2 with echelon-3 lymph node stations). The FDR of PET to detect EBUS-positive nodes rose significantly from 5.3% (95% CI: 1.7–8.8%) over 32.9% (95% CI: 25.9–40.0%) to 69.1% (95% CI: 56.9–81.3%) at echelons 1–3 (*p* < 0.0001, Fisher’s exact test for comparison of all three echelons as well as echelon-1 with echelon-2 or echelon-2 with echelon-3). The rate of EBUS-positive lymph nodes among PET-positive, EBUS-probed LNs is equal to (1 – FDR) for the respective echelons. The FDR of PET to detect truly involved lymph nodes can be estimated to 16.1 and 61.4% at echelons-2 and -3 according to Eq. 3, assuming a sensitivity of EBUS of 80%.

**Table 4:** PET- and EBUS- positivity / negativity per EBUS-probed LN in LN echelon-1, -2 and -3.

LN echelon-1	PET+:	*n *= 152	PET-:	*n* = 17
	PET+, EBUS+:	*n* = 144	PET-, EBUS+:	*n* = 1
LN echelon-2	PET+:	*n* = 170	PET-:	*n* = 127
	PET+, EBUS+:	*n* = 114	PET-, EBUS+:	*n* = 12
LN echelon-3	PET+:	*n* = 55	PET-:	*n =* 154
	PET+, EBUS+:	*n* = 17	PET-, EBUS+:	*n* = 3

Note. All numbers represent LN counts with the respective characteristics in the different LN echelons. A total of 675 LNs were EBUS-probed in the different LN chains

The sensitivity of PET in comparison to EBUS stayed above 80% with 99.3 (95% CI: 98.0–100%), 90.5 (95% CI: 85.4–95.6%), and 85.0% (95% CI: 69.4–100%) at echelon-1, -2, and -3, respectively (*p* < 0.0001, Fisher’s exact test comparing all three echelons as well as comparing pairwise the echelons). The specificity of PET in comparison to EBUS was 66.7 (95% CI: 47.8–85.5%), 67.3 (95% CI: 60.2–74.3%), and 79.9% (95% CI: 74.2–85.6%) at echelon-1, -2 and -3, respectively (*p* = 0.017, Fisher’s exact test comparing all three echelons, *p* = 0.002, comparing echelon-2 with echelon-3). PET-positive findings in echelon-3 LNs were graded as Likert 3 and 4 in 31 and 69% of cases in the nuclear medicine report, respectively.

Furthermore, the coverage of a LN-station by the clinical target volume was analyzed in dependence on EBUS- and PET-positivity in this station (Table 3). All but two EBUS-positive LN-stations in separate patients were included in the target volume. One of the two patients had primary lung cancers in both lungs: one primary was resected and another treated by definitive radiochemotherapy. The EBUS- and PET-positive hilar LNs contralateral to the irradiated primary tumor were resected and not included in the target volume. In the second patient, a PET-negative LN-station was not included as the patient was treated on a neoadjuvant radiochemotherapy schedule. From the 377 PET-positive LN-stations, 338 were included in the CTV (Table 3). Those 39 PET-positive LN-stations not included were EBUS-negative in 38 cases or upfront resected in 1 case.

The inclusion of a LN-station in the CTV was significantly dependent on both, PET- and EBUS-positivity, as main effects using logistic regression (*p* < 0.0001 for both factors, Wald test). The ratio of the odds of inclusion of a LN-station in the target volume was 106.7 (95% CI: 32.5–658.6) for EBUS-positive vs. EBUS-negative and 6.0 (95% CI: 3.7–9.9) for PET-positive vs. PET-negative LNs.

A total of 127 EBUS-negative LN-stations was covered by the CTV among the patients of this study (Table 3). Of these, 64 were PET-positive, and the remaining 63 PET-negative stations were included in the CTV based on the judgment of the treating physician. A considerable number of the latter were skipped LN-stations defined here as stations in-between involved stations, or overlapped stations, where at least in two axial slides with at least 1 cm cranio-caudal distance, the axial center of the LN-station was within 1 cm of an involved LN (*n* = 33). The odds of irradiated LN-stations among EBUS-negative LN-stations was higher for neoadjuvant compared with definitive radiochemotherapy (odds ratio = 1.79 (95% CI: 1.17–2.77); *p* = 0.008, Fisher’s exact test). This reflects the more generous inclusion of mediastinal lymphatics at risk to a lower total dose of trimodality treatment, as performed in all major trials on neoadjuvant radiochemotherapy [5, 18–20] and also in this study. Among the 161 EBUS- and PET-negative LN-stations in patients undergoing definitive radiochemotherapy, there were 25 irradiated LN-stations and 20 of them were skipped or overlapped stations (Table Suppl. 2a).

The actually treated target volumes in this study were changed on the basis of the EBUS findings in 40 patients (22.2% of all patients), in contrast to a contouring rule that included PET-positive LN-stations alone as a sufficient and necessary criterion [1, 2]. Fifteen PET-negative and EBUS-positive LN-stations were included in the actually treated target volumes in additional 12 patients. Moreover, 38 PET-positive and EBUS-negative LN-stations were not included in the CTV in 28 patients (15.5% of patients). The excluded LNs from the CTV were located in the contralateral hilum in 13 of the latter patients. At a median follow-up of 27 months till the last imaging examination, none of those 28 patients developed a relapse in these PET-positive and EBUS-negative LN-stations. Survival at 2 and 3 years was 61%. Fifteen of these 38 PET-positive and EBUS-negative lymph nodes, or particularly, 3 of the 15 PET-positive and EBUS-negative lymph nodes in echelon-3, received an incidental radiation dose greater than 40 Gy because they were too close to the target volume.

## Discussion

This study shows that the rate of EBUS-positive LN metastases in FDG-PET-positive nodes significantly decreases with distance from the primary tumor along the lymphatic drainage from echelon-1 to echelon-3. Accordingly, the FDR of FDG-PET compared with EBUS markedly increases from 5.3% in echelon-1 to 69.1% in echelon-3 LNs. We included consecutive patients with clinical stage III NSCLC elected for definitive or neoadjuvant radiochemotherapy. This study used systematic EBUS with a mean of 3.75 lymph node stations sampled per patient.

Previous studies comparing PET results with EBUS results or histopathologic findings after tumor resection predominately include lung cancer undergoing staging prior to surgery at earlier stages [8, 21–24]. In these retrospective studies, the fraction of patients with affected mediastinal lymph nodes was considerably lower than in the present study, ranging from 12 to 22%, whereas the fraction of patients with EBUS-positive mediastinal lymph nodes was 60% in this study. The unit of observation in the previous studies was the involvement of hilar and mediastinal lymph nodes per patient. None of these studies considered the dependence of prevalence of involved lymph nodes on distance of the lymph node station from the primary tumor. The FDR of PET/CT in these studies was about 45% at the lower prevalence of mediastinal involvement [21, 22, 24]. From the meta-analysis by Schmidt-Hansen et al., an estimate of the FDR for diagnosis of an N2 status per patient undergoing tumor resection can be derived, with the prevalence of 25% and FDR of 34% in over 6095 patients [8]. Gan et al. analyzed cytological results from EBUS-TBNA for PET-positivity LNs from the database of the MD Anderson Cancer Center [25]. Only the predominant PET-positive lymph node was analyzed in that study with a mean of 1.3 analyzed positive lymph nodes per patient. In 41% of their patients, EBUS-TBNA was performed for initial staging for lung cancer. In these patients, the FDR of [^18^F]FDG-PET was 31% compared with EBUS-TBNA as a reference, given the prevalence of 69% of patients with EBUS-positive nodes. PET-positive and cytology-negative lymph nodes showed lymphoid tissue only in 70%, granulomatous inflammation in 18%, anthracotic pigment-laden macrophages in 9%, and extensive necrosis with histiocytes in 2% of cases. As the FDR decreases with increasing prevalence for a given sensitivity and specificity, these studies are approximately consistent with the findings of the present study with an overall FDR of PET/CT of 27% at a prevalence of EBUS-positive N2 or N3 lymph nodes of 60% of patients. While all of these studies underline that the considerable false discovery rates of lymph node metastases limit the accuracy of FDG-PET/CT for predicting the involvement per lymph node station, the present study shows that the FDR of FDG-PET/CT increases with the distance of the lymph node station from the primary tumor.

To estimate the underlying true rate of involved lymph node stations from the rate of EBUS-positive lymph node stations, a specificity of nearly 100% and a sensitivity of 80% of EBUS-TBNA were assumed throughout the present study. This is supported by several prospective [26–32] and large retrospective studies [33, 34]. The SCORE study included 229 patients with known or suspected NSCLC [26]. The EBUS of PET/CT targets alone or in combination with systematic sampling had a sensitivity of 73% and 77%, respectively, for detecting mediastinal metastases in patients with mediastinal involvement. The addition of EUS increased sensitivity to 82%. In 11 of 19 patients with false-negative mediastinal staging by EBUS and EUS, LN metastases were located in LN-stations 5, 6 and 3A not accessible by EBUS/EUS, in 3 patients only micro-metastases were found by the reference test. Sanz-Santos et al. included patients with cN0/N1 NSCLC by EBUS-TBNA and compared the results with surgery [28]. EBUS was false-negative in 14% of cases, but only in 3% when systematic LN staging was performed. Thus, systematic EBUS-TBNA sampling was recommended. Kim et al. found a very low false-negative discovery rate in accessible LNs of 4.5% by combined EBUS-TBNA/EUS [35]. Recent meta-analyses on more than 2000 patients confirmed a sensitivity of EBUS-TBNA for mediastinal staging in the range of 80–92% in comparison to a surgical mediastinal evaluation on a per patient basis [36–40].

Two currently accepted consensus on radiotherapy planning recommend the inclusion of LNs that are FDG-PET-positive but EBUS-negative in the gross target volume [1, 2]. These recommendations are primarily based on the work of Peeters et al., who assumed a prevalence of cancer in 78 and 70% of enlarged and normal sized [^18^F]FDG-PET-positive LNs and a false-negative rate of EBUS-TBNA of 20% [41]. They concluded that PET-positive and EBUS-negative LNs should be included in the GTV because the prevalence of cancer in these nodes is about 14–16% [41]. Exceptions from this rule could be symmetrical bilateral PET-positive LNs with a nonmalignant diagnosis as silicosis or granulomatosis [41].

According to the results of the present study, the FDR of FDG-PET/CT increases with the distance of the lymph node station from the primary tumor. A positive PET-finding in an echelon-3 LN in the upper or contralateral mediastinum may significantly alter the target volume and with a FDR of greater than 50%, the number of false positive FDG-PET findings will be substantial. In their small study, Cole et al. also pointed that EBUS-TBNA in addition to PET/CT has the potential to reduce the target volume and treatment associated toxicity in 6 of their 30 patients [42]. According to the present study, the true prevalence of cancer in a PET-positive echelon-3 LN can be estimated to 40% in patients with stage III NSCLC selected for radiochemotherapy. Then, the probability of a truly involved PET-positive lymph node is 8% if EBUS is negative, assuming a sensitivity of the test of 80%, a rather low value for a uniform recommendation of inclusion. In fact, in the clinical routine, we omitted 43% of all PET-positive and EBUS-negative LNs from the clinical target volumes in definitive radiochemotherapy. Only a few studies directly detailed the prevalence of LN metastases from NSCLC in PET-positive and EBUS-negative LNs. Bouwens et al. found a low prevalence of 10% of LN metastases in PET-positive, EBUS-negative nodes, whereas the underlying prevalence in PET-positive LNs was 56% in that study [43].

A limitation of the present study is that the true prevalence of metastases in the lymph node echelon of interest was not available from upfront systematic lymph node dissection. This is the consequence of the fact that only patients with locally advanced NSCLC selected for definitive or neoadjuvant radiochemotherapy were included in this study. This group of patients had a higher prevalence of mediastinal lymph node metastases than studies of surgical patients [21–24]. This fact is most relevant for investigating the impact of EBUS-TBNA in addition to [^18^F]FDG-PET/CT imaging on radiotherapy target volume definition. Therefore, instead of estimating the FDR of PET compared with the true lymph node positivity, the FDR of PET was estimated compared with EBUS-positivity, assuming a sensitivity and specificity of EBUS-TBNA of 80 and 100%, respectively. In addition, clinical follow-up was available for patients with PET-positive lymph nodes which were not included in the target volume because they were EBUS-TBNA negative. However, no local recurrences were observed at these localizations.

The results of our study show that the inclusion of PET-positive and EBUS-negative LNs should be reconsidered according to the echelon of involvement, especially in echelon-2 and -3 LNs. In the case of PET-positive and EBUS-negative LNs, further diagnostic clarification is recommended in accordance with the ESGE/ERS/ESTS guideline [10]. EBUS in combination with EUS is suggested in the PET-positive mediastinum in patients with suspected or proven NSCLC. Subsequent surgical staging (VATS mediastinoscopy) might be recommended in selected cases, when EBUS-EUS does not show malignant nodal involvement [10]. Nasir et al. reviewed factors where negative EBUS should be confirmed by additional invasive procedures, i.e., mediastinoscopy or thoracoscopy [44]. They recommended additional invasive procedures in cases with a non-diagnostic EBUS result, i.e. the absence of lymphocytes, with a highly suspicious [^18^F]FDG-PET/CT scan. For left upper lobe tumors, they recommended separate sampling of LN-station 5/6, if other LNs are uninvolved by EBUS [44].

## Conclusions

In summary, the prevalence of LN involvement in PET-positive nodes decreases markedly with distance from the primary tumor along the lymphatic chains from the ipsilateral hilum to the upper or contralateral mediastinum or contralateral hilum in a stage III NSCLC radiochemotherapy cohort. This leads to a steep increase in the false discovery rate by PET-CT in echelon-3 LNs. EBUS-TBNA with almost 100% specificity and good sensitivity is recommended especially for the diagnostic clarification of PET-positive LNs in more distant LN-stations. The inclusion of these would have a major influence on the size of the target volume. In fact, in 22.2% of patients in this series, the target volume was altered by EBUS-TBNA compared to a PET-based delineation alone.

## Supplementary Information

ESM 1 (DOCX 22 kb)

## Data Availability

All data generated and analyzed during this study are included in this published article (supplementary information can be received from the corresponding author).
