# Supplementary material for: Impact of EBUS-TBNA in addition to [18F]FDG-PET/CT imaging on target volume definition for radiochemotherapy in stage III NSCLC
Source: Eur J Nucl Med Mol Imaging. 2021 Feb 5;48(9):2894–903. doi: 10.1007/s00259-021-05204-7 (PMC8263445; doi:10.1007/s00259-021-05204-7)
Supplement: Supplementary file 1 — (DOCX 22 kb) [file 259_2021_5204_MOESM1_ESM.docx]

**Table Suppl. 2a** Cross tabulation of results from EBUS-TBNA, PET-reports, and target volume coverage analysis from patients with locally advanced NSCLC, stage IIIA-IIIC, treated with definitive radiochemotherapy. *N* = 436 LN-stations in 114 patients

|  | **Nuclear medicine report positive** | **Nuclear medicine report negative** | **Irradiated LN-stations** | **Not-irradiated LN-stations** |
| --- | --- | --- | --- | --- |
| EBUS-TBNA positive | 196 | 10 | 205 | 1 |
| EBUS-TBNA negative | 69 | 161 | 64 | 166 |
| Irradiated LN-station | 234 | 35 |  |  |
| Not-irradiated LN-station | 31 | 136 |  |  |
| Nuclear medicine report positive and EBUS-negative LN-stations |  |  | 39 | 30 |
| Nuclear medicine report negative and EBUS-positive LN-stations |  |  | 10 | 0 |
| Nuclear medicine report negative and EBUS-negative LN-stations |  |  | 25 | 136 |

Note: All numbers represent lymph node station (LN-station) counts
